# Hypertriglyceridemia-Induced Acute Pancreatitis During Pregnancy: A Case Report

**DOI:** 10.7759/cureus.28273

**Published:** 2022-08-22

**Authors:** Darian Keller, Ellen M Hardin, Sai V Nagula, Anthony Royek

**Affiliations:** 1 Internal Medicine, Mercer University School of Medicine, Savannah, USA; 2 Obstetrics and Gynecology, Mercer University School of Medicine, Savannah, USA; 3 Obstetrics and Gynecology, Memorial Health University Medical Center, Savannah, USA

**Keywords:** hypertrygliceridemic pancreatitis, pancreatitis in pregnancy, idiopathic pancreatitis in pregnancy, severe hypertriglyceridemia, hypertriglyceridemia-induced acute pancreatitis

## Abstract

Hypertriglyceridemia-induced acute pancreatitis is a rare and serious condition that places both the mother and the fetus at severe risk for morbidity and mortality. The goal of this case report is to describe the management of a pregnant patient with severely elevated triglycerides in the setting of acute pancreatitis. A 28-year-old female G2P1001 at 29 weeks of gestational age presented with epigastric abdominal pain. A computed tomography scan of the abdomen and pelvis with contrast demonstrated acute interstitial edematous pancreatitis. A lipid panel was performed, revealing a serum triglyceride level of 3,949 mg/dL. Insulin and maternal bowel rest reduced her serum triglyceride levels; however, additional medical therapy including fibrate and statin drugs were initiated to achieve goal levels of triglycerides and improve patient symptoms. The patient ultimately recovered and remained on treatment until delivery. Initial management addresses acute pancreatitis and involves fluid resuscitation, pain control, and bowel rest. Triglyceride-lowering drug therapies are rarely used during pregnancy due to the potential for fetal teratogenicity; however, given the severity of hypertriglyceridemia fenofibrate and atorvastatin were prescribed. Additional medical treatment included insulin, omega-3, and ethyl eicosapentaenoic acid.

## Introduction

Acute pancreatitis during pregnancy is a severe condition that puts both the mother and the fetus at risk for complications and mortality. Hypertriglyceridemia is one of the most common causes of acute pancreatitis and accounts for up to 56% of pancreatitis cases during pregnancy [1]. The degree of triglyceride elevation correlates with the risk of developing acute pancreatitis. There is a 5% risk for acute pancreatitis with triglyceride levels greater than 1,000 mg/dL, and a 10-20% risk with levels greater than 2,000 mg/dL [2]. The risk for acute pancreatitis also increases with the number of previous episodes of pancreatitis [3]. Triglycerides are broken down by pancreatic lipases into free fatty acids. These free fatty acids are lipotoxic and, in addition to the inflammatory response to pancreatitis itself, contribute to the severity of acute pancreatitis [4]. Management of hypertriglyceridemia-induced acute pancreatitis involves supportive treatment with intravenous (IV) fluids and pain control and reduction of triglyceride levels [5].

## Case presentation

A 28-year-old female Gravida 2, Para 1, at 29 weeks and one day presented as a transfer from an outside hospital for abdominal pain which began the previous morning. Her previous pregnancy was complicated by cesarean section for non-reassuring fetal heart tones, and she reported a history of pancreatitis a year prior. Her pain was constant and generalized across the entire abdomen and worse in the epigastrium. She had associated nausea and vomiting beginning the previous day. She reported shortness of breath and chest pain on admission.

On physical examination, she appeared in moderate distress. She was febrile (100.6­­°F), tachycardic, and tachypneic. Her ­abdomen was gravid, exquisitely tender to palpation over the epigastrium, right upper quadrant, and left upper quadrant. There was moderate tenderness to palpation over the lower quadrants. The fetal assessment showed a baseline of 160, moderate variability, accelerations, and no decelerations. Ultrasound at an outside hospital showed diffuse hepatic steatosis, biliary duct dilation, and gallbladder distention without wall thickening or cholelithiasis. Labs from the outside hospital showed an elevated white blood cell count (14.8 K/µL) mildly elevated amylase (157 U/L) and lipase (138 U/L) (Table 1).

**Table 1:** Relevant laboratory investigations. WBC: white blood cell

	Outside hospital	On admission	Hospital day 2	Hospital day 3	Hospital day 13
WBC (reference: 3.6–11.0 K/µL)	14.6 K/µL	14.6 K/µL	16.7 K/µL	13.9 K/µL	
Amylase (reference: 25–115 U/L)	157 U/L	73 U/L			
Lipase (reference: 10–140 U/L)	138 U/L	286 U/L			
Triglycerides (reference: <150 mg/dL)				3,949 mg/dL	953 mg/dL

**Figure 1:**
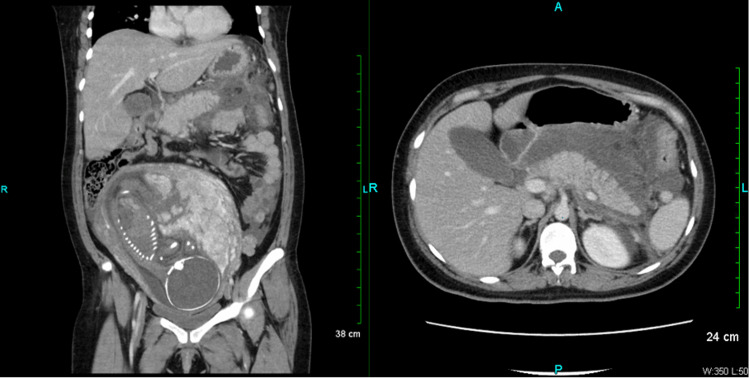
Coronal section (left) and axial section (right). Computed tomography scan of the abdomen and pelvis with contrast showing acute interstitial edematous pancreatitis with extensive peripancreatic fluid extending throughout the upper abdomen. No evidence of pancreatic necrosis.

She started on aggressive IV fluids, NPO, and morphine as needed for pain. A computed tomography (CT) of the abdomen and pelvis with contrast revealed acute interstitial edematous pancreatitis with extensive peripancreatic fluid extending throughout the upper abdomen, and no evidence of pancreatic necrosis or biliary duct dilatation (Figure 1). Her white blood cell count was 14.6 K/µL and amylase and lipase were 73 U/L and 286 U/L, respectively.

She was doing well on day two of hospitalization until the evening when she become febrile (101.4°F) and tachycardic (125 beats per minute). She was flagged for sepsis with an unclear etiology. Her white blood cell count was 16.7 K/µL, urinalysis was unremarkable, and a chest X-ray was notable for consolidation and atelectasis resulting from pneumonia or congestion. Blood cultures were ordered, but they would ultimately be negative for growth. She was started empirically on IV vancomycin and Zosyn.

The following day, the patient reported marked symptom improvement, but she continued to experience epigastric pain. Her lab work was significant for a decrease in white blood cell count (13.9 K/µL) and markedly elevated triglycerides (3,949 mg/dL). After careful consideration, she was started on two units of IV insulin per hour (0.03 units/kg/hour) and D10W-1/2 NS (100 mL/hour), with the goal of decreasing her triglyceride level to less than 1,000 mg/dL, thereby decreasing her risk of recurrent, life-threatening pancreatitis. Frequent blood glucose monitoring was initiated to ensure she did not develop hypoglycemia.

On hospital day seven, her pain had resolved and her insulin therapy was discontinued, but her triglyceride level remained slightly above 1,000 mg/dL. She was started on fenofibrate, omega-3, and ethyl eicosapentaenoic acid. Unfortunately, her triglycerides remained elevated and atorvastatin 20 mg was initiated after informed consent. Nephrology was about to be consulted for plasmapheresis, but the goal triglyceride level was reached prior to their evaluation. After continued resolution of pain and a triglyceride level of 953 mg/dL, the patient was discharged home with plans for outpatient follow-up. The patient gave informed consent for the publication of this case report.

## Discussion

Pregnancy is known to cause an elevation in triglyceride levels, particularly during the third trimester. However, the total serum triglyceride level rarely exceeds 300 mg/dL and is unlikely to cause acute pancreatitis on its own. More often, hypertriglyceridemia-induced acute pancreatitis occurring during pregnancy has an underlying genetic cause [6]. This patient had no known history of familial hypertriglyceridemia or dyslipidemias. No genetic testing was conducted on this patient to elucidate the cause of her hypertriglyceridemia, but that may have proved interesting from an academic perspective.

Hypertriglyceridemia-induced acute pancreatitis during pregnancy is rare, and little evidence-based research is available to guide care. Management relies on a multidisciplinary care team and physician experience. Initial management addresses acute pancreatitis and involves fluid resuscitation, pain control, and bowel rest [5]. Once hypertriglyceridemia has been identified, therapies should be initiated to lower serum triglycerides. The therapies used in this case included insulin, fenofibrate, omega-3, ethyl eicosapentaenoic acid, and atorvastatin.

Insulin enhances lipoprotein lipase (LPL) activity, thereby decreasing the production of very-low-density lipoprotein and lowering serum triglycerides [7]. In this patient, a low dose of insulin was given (0.03 U/kg/hour), and D10W-1/2 NS (100 mL/hour) was used to avoid hypoglycemia. There is little evidence that insulin infusion is superior in lowering serum triglycerides when compared to an NPO diet in the management of hypertriglyceridemia-induced acute pancreatitis [8].

Fibrates activate peroxisome proliferator-activated receptor α, leading to increased transcription of proteins involved in the metabolism of triglycerides [9]. In patients with a genetic cause of hypertriglyceridemia, fibrates can effectively lower serum triglycerides in a manner dependent on the patient’s particular genotype [10]. Omega-3 ethyl esters (including ethyl eicosapentaenoic acid) downregulate hepatic lipogenesis and upregulate fatty acid oxidation in the liver and skeletal muscle [11].

The Food and Drug Administration recommends that most statin use be discontinued during pregnancy. However, statins may still be indicated in certain situations where the benefit outweighs the risk. The FDA recommends a thorough discussion between the physician and patient before continuing or initiating therapy during pregnancy [12].

## Conclusions

This patient developed acute pancreatitis during pregnancy as a result of remarkably elevated triglycerides (~4,000 mg/dL). Insulin and bowel rest reduced her serum triglyceride level significantly, but her serum triglycerides still exceeded 1,000 mg/dL. Her care team employed various additional therapies to achieve goal triglyceride levels. By this time, the patient had been free of pain for several days and was discharged with close outpatient follow-up. This case report illustrates the presentation and management of a rarely seen condition.

## References

[1] Chang CC, Hsieh YY, Tsai HD, Yang TC, Yeh LS, Hsu TY (1998). Acute pancreatitis in pregnancy. Zhonghua Yi Xue Za Zhi (Taipei).

[2] Scherer J, Singh VP, Pitchumoni CS, Yadav D (2014). Issues in hypertriglyceridemic pancreatitis: an update. J Clin Gastroenterol.

[3] Sanchez RJ, Ge W, Wei W, Ponda MP, Rosenson RS (2021). The association of triglyceride levels with the incidence of initial and recurrent acute pancreatitis. Lipids Health Dis.

[4] Yang F, Wang Y, Sternfeld L (2009). The role of free fatty acids, pancreatic lipase and Ca+ signalling in injury of isolated acinar cells and pancreatitis model in lipoprotein lipase-deficient mice. Acta Physiol (Oxf).

[5] Cruciat G, Nemeti G, Goidescu I, Anitan S, Florian A (2020). Hypertriglyceridemia triggered acute pancreatitis in pregnancy - diagnostic approach, management and follow-up care. Lipids Health Dis.

[6] Eskandar O, Eckford S, Roberts TL (2007). Severe, gestational, non-familial, non-genetic hypertriglyceridemia. J Obstet Gynaecol Res.

[7] Eckel RH (1989). Lipoprotein lipase. A multifunctional enzyme relevant to common metabolic diseases. N Engl J Med.

[8] Berberich AJ, Ziada A, Zou GY, Hegele RA (2019). Conservative management in hypertriglyceridemia-associated pancreatitis. J Intern Med.

[9] Staels B, Dallongeville J, Auwerx J, Schoonjans K, Leitersdorf E, Fruchart JC (1998). Mechanism of action of fibrates on lipid and lipoprotein metabolism. Circulation.

[10] Brisson D, Ledoux K, Bossé Y (2002). Effect of apolipoprotein E, peroxisome proliferator-activated receptor alpha and lipoprotein lipase gene mutations on the ability of fenofibrate to improve lipid profiles and reach clinical guideline targets among hypertriglyceridemic patients. Pharmacogenetics.

[11] Davidson MH (2006). Mechanisms for the hypotriglyceridemic effect of marine omega-3 fatty acids. Am J Cardiol.

[12] (2021). US Food and Drug Administration. FDA requests removal of strongest warning against using cholesterol-lowering statins during pregnancy; still advises most pregnant patients should stop taking statins. https://www.fda.gov/drugs/fda-drug-safety-podcasts/fda-requests-removal-strongest-warning-against-using-cholesterol-lowering-statins-during-pregnancy.

